# Complete genome sequence of *Arcanobacterium wilhelmae* strain DSM 102162 isolated from the genital tract of a *Rhinoceros unicornis*

**DOI:** 10.1128/mra.00204-24

**Published:** 2024-07-18

**Authors:** Maria Borowiaka, Antonia Kreitlow, Burkhard Malorny, Christoph Lämmler, Jörg Rau, Madeleine Plötz, Amir Abdulmawjood

**Affiliations:** 1 Department for Biological Safety, German Federal Institute for Risk Assessment (BfR), Berlin, Germany; 2 Institute of Food Quality and Food Safety, University of Veterinary Medicine Hannover, Hannover, Germany; 3 Institut für Hygiene und Infektionskrankheiten der Tiere, Justus-Liebig-Universität Gießen, Gießen, Germany; 4 Chemisches und Veterinärunterschungsamt Stuttgart (CVUAS), Fellbach, Germany; Rochester Institute of Technology, Rochester, New York, USA

**Keywords:** *Arcanobacterium wilhelmae*, *Rhinoceros unicornis*

## Abstract

Previous case reports indicate *Arcanobacterium*’s opportunistic pathogenic potential. However, the true diversity of the genus remains understudied. Here, we present the complete genome of *Arcanobacterium wilhelmae* isolated from a diseased rhinoceros, suspected to play a role in its condition. These genomic data may enable future advancements in understanding *Arcanobacterium* pathogenicity.

## ANNOUNCEMENT

*Arcanobacterium* is a genus of Gram-positive, non-motile, facultative anaerobic bacteria commonly isolated from animal and human samples (1). Usually, *Arcanobacterium* exists as part of the normal flora of skin and mucous membranes (2). However, previous studies support that some representatives of the genus possess an opportunistic pathogenic potential and are able to cause severe infections, especially when the host suffers from immunosuppressive conditions (3). Examples have included *Arcanobacterium haemolyticus*, causing wound infections and pharyngitis in humans (4); *Arcanobacterium canis*, associated with otitis externa in a dog (5); and *Arcanobacterium phocae*, isolated from inflammation tissue sides from stranded marine mammals (6). The pathogenic potential of bacteria belonging to the *Arcanobacterium* genus and their interactions with other pathogens are generally not well understood (4).

In this study, we present the complete genome sequence of *A. wilhelmae* strain DSM 102162, isolated in 2012 in association with other potentially pathogenic bacteria (*Streptococcus* sp. and *Escherichia coli*) from a vaginal swab of an Asian rhinoceros (*Rhinoceros unicornis*) at Wilhelma Zoo and Botanical Garden, Stuttgart, Germany. The rhinoceros displayed fertility problems and vaginal discharge, raising intriguing questions about the potential role of *Arcanobacterium wilhelmae* in these conditions (7, 8).

The strain was retrieved from the in-house cryo-culture collection and cultivated under microaerobic conditions on sheep blood agar for 48 h at 37°C. A single genomic DNA extract was prepared using the PureLink Genomic DNA Mini kit (Thermo Fisher Scientific) following the manufacturer’s instructions and subjected to Illumina and Oxford Nanopore Technologies (ONT) sequencing and subsequent data analysis (9). Default parameters were used for all software mentioned in the following.

An Illumina sequencing library was prepared using the Illumina DNA Prep (M) Tagmentation kit and sequenced in 2×  301-bp cycles on an Illumina MiSeq sequencer (MiSeq Reagent Kit v.3). Generated short reads were trimmed using fastp v.0.22.0 (10), resulting in 0.6 million high-quality paired-end reads (≥79.1% Q30).

An ONT sequencing library was prepared using the SQK-RBK110.96 kit and sequenced on a Minion Mk1C device using an R9.4.1 (FLO-MIN106) flow cell. Generated fast5 data were subsequently basecalled using ONT guppy v.6.0.1 (https://community.nanoporetech.com/downloads) in super accuracy (SUP) mode on a graphics processing unit server. The obtained fastq data were trimmed, filtered, and quality-checked using Porechop v.0.2.4 (https://github.com/rrwick/Porechop), NanoFilt v.2.8.0, and NanoStat v.1.5.0 (11), resulting in 8,788 filtered reads (read length *N*_50_ value: 5,395 bp, mean read quality score: 13.7).

Illumina and ONT data sets were subjected to *de novo* hybrid assembly via Unicycler v.0.4.8 (12 – 14). The resulting assembly included one circularized chromosome of 2,015,622  bp with a G + C content of 60.7% (determined in Geneious Prime v.2020.2.2) and was annotated using PGAP v.6.5 (15).

**Fig 1:**
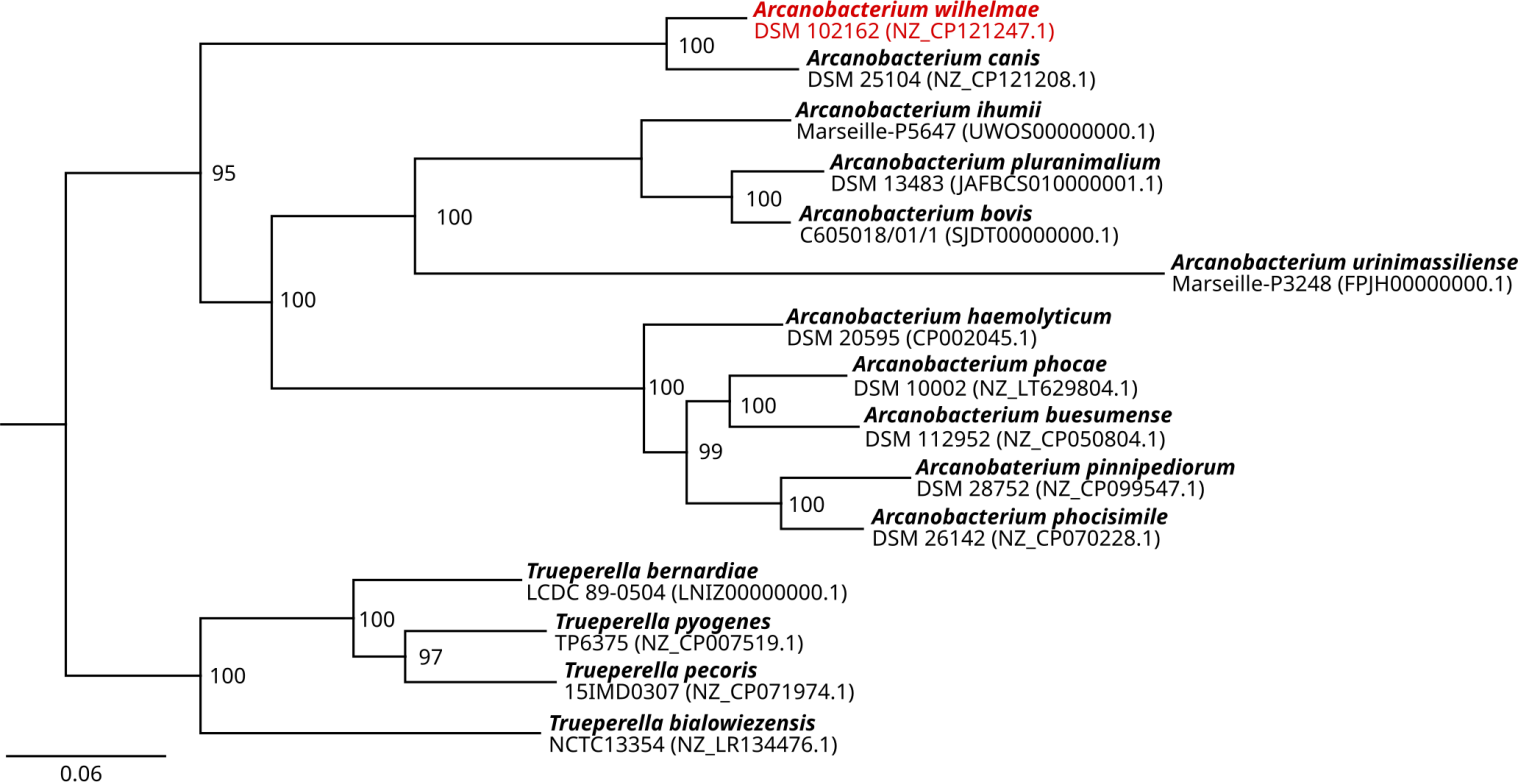
Maximum likelihood phylogenetic tree illustrating the phylogenetic relationship between *Arcanobacterium wilhelmae* and other species within the *Arcanobacterium* genus, alongside closely related representatives from the *Truperella* genus. The tree was constructed using bcgtree v.1.2.0 and is based on the alignment of the amino acid sequence of 107 core genes (16). The numbers shown at the branches represent the bootstrap support values obtained from 100 bootstrap replicates. The scale bar represents the number of amino acid substitutions per site.

For phylogenetic comparison with previously reported *Arcanobacterium* and closely related *Truperella* species, a comparison of the amino acid sequence of 107 core genes was conducted using bcgTree v.1.2.0 (16) on the basis of available representative genome assemblies from National Center for Biotechnology Information. Assemblies were uniformly annotated with Prokka v.1.14.0 (17); generated .faa files were subjected to bcgTree; and the resulting maximum likelihood tree was visualized in Geneious Prime v.2020.2.2, rooted to the *Truperella* outgroup, and finalized in InkScape v.1.3 (Fig. 1). *A. wilhelmae* clusters closely with *A. canis*, another *Arcanobacterium* species suspected of pathogenic potential. This underscores the importance of further investigating the pathogenic capabilities of the *Arcanobacterium* genus.

## Data Availability

The sequencing read data sets {ONT [Sequence Read Archive (SRA): SRX19820730] and Illumina [SRA: SRX19820729]}, as well as the chromosome (GenBank: CP121247.1) of Arcanobacterium wilhelmae, are deposited in the National Center for Biotechnology Information database under BioProject PRJNA950366/BioSample SAMN33987071.
